# A Plea for the Education of Feeble-Minded Children

**Published:** 1882-11

**Authors:** 


### Editorial.

### A PLEA FOR THE EDUCATION OF FEEBLE-MINDED CHILDREN.

Perfect mental health, like perfect physical health, is beyond the hope of humanity. A mind exactly and equally developed in all of its parts, exhibiting a minutely balanced relation between the several faculties that make up the whole, is scarcely to be found among men in their fallen estate.

Nevertheless, when the sub-forces of the mind—the perception, intellect, emotion and will—act co-ordinately and harmoniously, and do not exhibit notable deviation from an average standard, either singly or collectively, the individual is justly considered sane.

Between this point and conspicuously declared insanity all varieties and degrees of mental alienation may be found. Illusions, hallucinations, delusions, defective memory and deficient reasoning power, varying degrees of exaltation and depression, and unresisted, if not irresistible impulse or a pitiable state of weakness, vacillation and indecision are often encountered in medical practice. So, in regard to original or early acquired defect. In the first years of childhood a wide deflection from what is accepted as normal intellectual force is frequently observed, when the child cannot properly be considered an idiot; for, as has just been implied, all shades of mental debility are found between the extremes which represent the strongest and the weakest, and those unfortunate beings who occupy these intermediate positions, who are not bright on the one hand or idiot on the other, offer every encouragement for intelligent and pains-taking training. It is for this class more particularly that we put forth this plea, and especially in their behalf do we invite the attention and invoke the active interest of our readers.

While this is true, extremely few, even of the most pronounced types of imbecility, are absolutely beyond the reach of properly directed effort, and to abandon any human being to hopeless mental and moral darkness without an earnest attempt to rescue him, is inexcusable, barbarous, inhuman; for it has been demonstrated that even the least promising subjects are ofttimes susceptible of an astonishing amount of improvement.

There is, in our opinion, no more inviting field for the exercise of true benevolence than the education—the mental development—of these unhappy little creatures. If they are left to struggle along with other children of stronger mental constitutions, with whom the daily contests are as hopeless as the prize fight between a giant and a dwarf, or between the trained athlete and the confirmed invalid, discouragement succeeds discouragement, defeat follows defeat, until finally a paralysing despair settles down upon the dimly lighted little mind and the long, desolate, gloomy night soon consumes the brief and flickering twilight.

Being deeply impressed with the importance of this subject, we believe that the noble and powerful energies of the state could not be directed into any channel which promises greater good or more abundant returns upon the labor and treasure which would be required, than the establishment and maintenance of a training school for feeble-minded children. This would be a practical charity, and we doubt not the suggestion will meet a responsive throb in the heart of every humanitarian within the bounds of the commonwealth.

This act upon the part of the State would be the highest evidence she could give of an advancing civilization, a growing public enlightenment and a pervading christian sentiment.

The idea is by no means novel, and the movement need not be an experiment. Such institutions are already in successful operation in many parts of the world, and there now lies before us the circular announcement of the Pennsylvania Training School for Feeble-Minded Children which was founded in 1853 and which has been sustained since that date by the fostering care of that progressive

State. Who can estimate the good that this single training school has wrought? Who can reckon the value of the little lives which it has rescued from perpetual darkness, or the amount of anguish it has averted? Who can know the full measure of the joy it has dispensed and the number of happy homes it has saved?

All this is well, and this country is to be congratulated that it is not entirely deprived of the benefits which flow from these institutions. But the schools now established are, from the nature of the case, accessible only to the few and the wealthy. The great mass of the citizens of this community are excluded from them both on account of the distances from their homes and the expense which this and other attendant circumstances involve.

Our space will not permit us to offer a plan in detail upon which one of these schools can be started, or to present elaborate arguments in favor of the bare suggestion which we have made, but it has occurred to us that an institution established after the general plan of the State Academy for the Blind, at Macon, would meet the present requirements and be a permanent ornament to the State.

We sincerely hope that some capable philanthropist will interest himself in this worthy cause and prosecute it to a practical issue.

